# 4862F, a New Inhibitor of HIV-1 Protease, from the Culture of *Streptomyces* I03A-04862

**DOI:** 10.3390/molecules18010236

**Published:** 2012-12-27

**Authors:** Xiao Liu, Maoluo Gan, Biao Dong, Tian Zhang, Yongzhen Li, Yuqin Zhang, Xiuyong Fan, Yexiang Wu, Shuoke Bai, Minghua Chen, Liyan Yu, Peizhen Tao, Wei Jiang, Shuyi Si

**Affiliations:** 1 Institute of Medicinal Biotechnology, Peking Union Medical College, Chinese Academy of Medical Sciences, Tiantanxili No.1, Beijing 100050, China; E-Mail: liuxiao503@gmail.com (X.L.); 2 National Institute of Biological Sciences, 7 Science Park Road, Zhongguancun Life Science Park, Beijing 102206, China

**Keywords:** *Streptomyces albosporus*, structure elucidation, HIV-1 protease inhibitor

## Abstract

We have isolated an extraordinary pentapeptide, called 4862F, from the culture broth of *Streptomyces albosporus* I03A-04862 by Diaion HP-20 macroporous adsorbent resin column, ODS-A and Sephadex LH-20 chromatography, followed by preparative HPLC. This peptide shows inhibitory activity against HIV-1 protease. The structure was elucidated by spectroscopic approaches, including ESI-MS and various NMR methods. Absolute configuration of the amino acid residues in 4862F was defined using Marfey’s method, and the structure was identified as *N,N,N-*(trimethylated)-Tyr-L-Leu-L-Val-L-Leu-(dehydrated)-His. The peptide 4862F displays inhibitory activity against HIV-1 protease, with IC_50_ values of 15.26 nM, using a fluorescence-based assay.

## 1. Introduction

The protease of human immunodeficiency virus type 1 is an essential enzyme in the viral life cycle where it cleaves the viral Gag-Pol polyprotein precursor into Gag proteins and two enzymes, integrase and protease [1]. Inhibition of this enzyme has been used successfully for the treatment of HIV-1-infected patients. Some HIV protease inhibitors, such as saquinavir, indinavir, and ritonavir, have been widely used clinically for the treatment of acquired Immunodeficiency syndrome (AIDS) patients. However, long-term use of these protease inhibitors leads to significant adverse effects and drug resistance. Therefore, it is imperative to find novel HIV-1 protease inhibitors.

Microbial fermentation products are important resources for the discovery of leading compounds with new structure and bioactivity. We are interested in the isolation of new inhibitors of HIV-1 protease, and the microbial products from an actinomycete strain *Streptomyces albosporus* I03A-04862 exhibited promising activity. We found that 1 mg·mL^−1^ of fermentation product (dried powder) from I03A-04862 could inhibit 50.2% of the activity of HIV-1 protease. This strain was classified as a member of the *Streptomyces* genus on the basis of 16S rRNA sequence analysis. A new pentapeptide has been isolated from the fermentation broth of this strain using a combination of Diaion HP-20 macroporous adsorbent resin column, ODS-A and Sephadex LH-20 chromatgraphy and preparative HPLC. Here we report the isolation and structure elucidation of this new compound. We also provide evidence for the demonstration of its inhibitory activity against HIV-1 protease *in vitro*.

## 2. Results and Discussion

**Figure 1:**
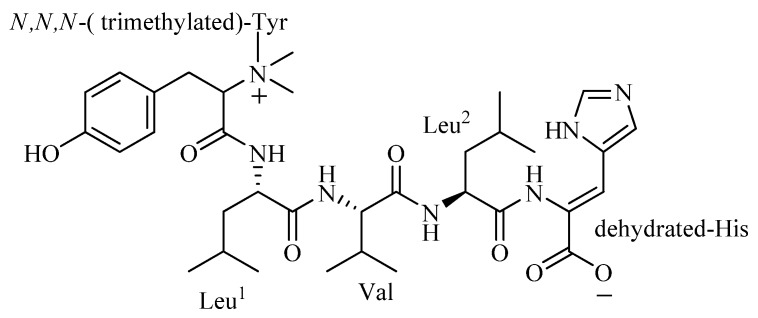
The structure of 4862F.

The compound 4862F (Figure 1) was isolated as a white amorphous powder and its molecular formula was established as C_35_H_53_N_7_O_7_ according to the HR-ESI(+) mass spectra, where the [M+H]^+^ ion was observed at *m/z* 684.40954 and [M+2H]^2+^ was observed at *m/z* 342.70860 (calcd for C_35_H_54_N_7_O_7_ 684.40847), [*α*]^20^_D_ +18° (MeOH, c 0.1).

The ^13^C-NMR and DEPT spectra of 4862F (Table 1) showed a total of 35 carbon signals, consisting of nine methyl groups including three *N*-methyls, three methylenes, fourteen methines, and nine quaternary carbons including five carboxyls. Interpretation of ^1^H, ^13^C, ^1^H-^1^H COSY, HSQC, and HMBC data of 4862F established the amino acid residues as leucine (Leu, 2×), valine (Val), and tyrosine (Tyr). In the HMBC spectrum, the *N*-CH_3_ signal (*δ*_C_ 53.255) showed a cross-peak to the *α*-H signal of Tyr (*δ*_H_ 4.046). The ^1^H-NMR spectra in D_2_O and 2% *d*-TFA showed nine protons (*δ*_H_ 3.128) covered up by the solvent peak when CD_3_OD was used as the solvent, which were correlated to the *N*-CH_3_ signal (δ_C_ 53.255) in the HSQC. This evidence indicates that the N of the Tyr residue was substituted by three methyls. The residue peaks suggests the presence of a histidine (His), which was dehydrated.

**Figure 2:**
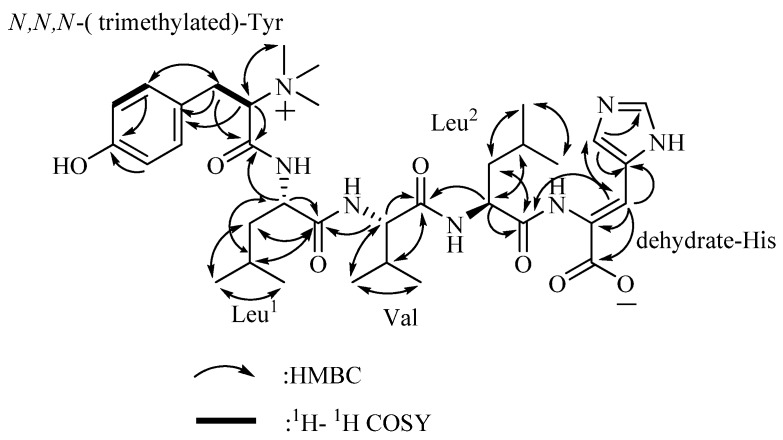
Selected 2D NMR correlations for 4862F.

The amino acid sequence of 4862F was analyzed using the HMBC correlations (Table 1, Figure 2) from relevant *α*-H to neighboring carboxylic carbons. The Leu^1^*α*-H (*δ*_H_ 4.363) signal showed an HMBC correlation to the amide carbon signal of trimethylated-Tyr (*δ*_C_ 166.550), indicating that it is acylated by trimethylated-Tyr. In the HMBC spectrum, the *α*-H signal of Val (*δ*_H_ 4.005) showed a cross-peak to the amide carbon signal of Leu^1^ (*δ*_C_ 172.756), the *α*-H signal of Leu^2^ (*δ*_H_ 4.335) showed a cross-peak to the amide carbon signal of Val (*δ*_C_ 174.520) and the *β*-H signal of the dehydrated-His (*δ*_H_ 4.335) showed a cross-peak to the amide carbon signal of Leu^2^ (*δ*_C_ 174.101). These correlations enabled assignment of the linear sequence for 4862F.

**Table 1:** NMR Spectroscopic Data of 4862F in CD_3_OD.

Unit	Pos.	δC *^a^*, mult.	δH *^b^* (*J* in Hz)	HMBC (H→C)
*N*,*N*,*N*-trimethyl-Tyr	CO	166.550, qC		
	*α*	77.147, CH	4.046, dd(11, 4)	CO, β, 1, N-Me
	*β*	32.979, CH_2_	3.139, dd(13, 11)	CO, 1, 2/6, *α*
			3.257, m *^c^*	
	1	125.257, qC		
	2/6	116.909, CH	6.967, d(8.5)	β, 3/5, 4
	3/5	131.441, CH	6.642, d(8.5)	1, 4
	4	158.085, qC		
	*N*-Me	53.255, 3*CH_3_	3.234, s	*α*
Leu^1^	CO	172.756, qC		
	*α*	53.357, CH	4.363, m *^c^*	CO, CO(Tyr), *β*, *γ*
	*β*	42.091, CH_2_	1.430, m *^c^*	CO, *α, γ, δ, δ'*
	*γ*	25.918, CH	1.388, m *^c^*	CO, *α, β, δ, δ'*
	*δ*	23.177, CH_3_	0.828, d(6.5)	*α, β, γ, δ'*
	*δ’*	22.319, CH_3_	0.806, d(6)	*α, β, γ, δ*
Val	CO	174.520, qC		
	*α*	60.443, CH	4.005, d(8)	CO, CO(Leu^1^), *β, γ, γ'*
	*β*	31.630,CH	1.991, m *^c^*	CO, *α, γ, γ'*
	*γ*	19.623, CH_3_	0.877, m *^c^*	*α, β, γ*
	*γ'*	19.351, CH_3_	0.916, m *^c^*	*α, β, γ'*
Leu^2^	CO	174.101, qC		
	*α*	54.110, CH	4.335, m *^c^*	CO, CO(Val), *β, γ*
	*β*	40.700, CH_2_	1.657, dd(7.5, 7.5)	CO, *α, γ, δ, δ'*
	*γ*	25.681, CH	1.760, m *^c^*	*α, β, δ, δ'*
	*δ*	23.405, CH_3_	0.936, d(7)	*β, γ, δ*
	*δ'*	21.887, CH_3_	0.884, d(6.5)	*β, γ, δ'*
(-2 *H*)-His	CO	166.164, qC		
	*α*	128.992, qC		
	*β*	119.794, CH	7.409, s	CO, CO(Leu^2^), 1,5, *α*
	1	128.745, qC		
	3	136.069, CH	8.902, s	5, 1
	5	122.783, CH	7.882, s	*β*, 1, 3

*^a^* Recorded at 125 MHz. *^b^* Recorded at 500 MHz. *^c^* Multiplicity due to overlapping.

**Figure 3:**
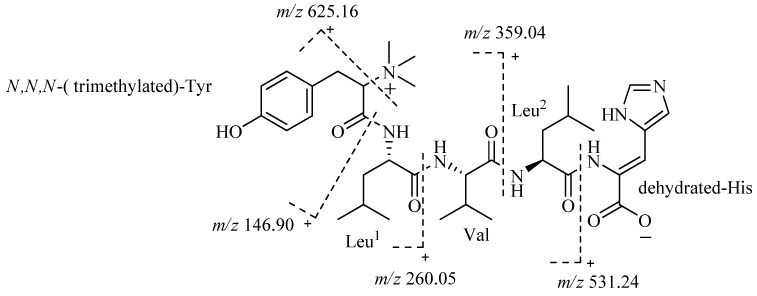
ESI-MS/MS data of 4862F.

The whole structure of 4862 F was further confirmed by analysis of ESI-MS/MS spectra (Figure 3). The fragment ion *m/z* 625.16 also confirmed that the *N* of Tyr is trimethylated. The fragment ion *m/z* 531.24 demonstrated that the amino acid residue of the C-terminal is a dehydrated-His.

Marfey’s method [2,3] was employed to assign the absolute configurations of the amino acid residues resulting from acid hydrolysis of 4862F. The 1-fluoro-2,4-dinitrophenyl-5-L-alanine amide (FDAA) derivatives of the acid hydrolysate of 4862F and the authentic D- and L-amino acids were subjected to HPLC analysis. The absolute configurations of all amino acid residues in 4862F except for trimethylated-Tyr and dehydrated-His were established by comparing their HPLC retention times with those of the corresponding authentic D- and L-standards. The results indicate that all the amino acid residues have L-configuration (Table 2).

**Table 2:** HPLC Analysis of FDAA Derivatized Acid Hydrolysates of Compound 4862F.

Amino acid	*t*R (min)	*t*R (min) of FDAA derivatized acid hydrolysates of compound 4862F
L-Leu	32.335	32.491
D-Leu	38.252	
L-Val	26.959	26.527
D-Val	32.950	

However, the determination of the absolute configuration of the trimethylated-Tyr using Marfey’s method and the *Z*, *E*- configuration of the trimethylated-Tyr residue was unsuccessful, because commercial standard materials are not available for these two residues.

To our knowledge, the structure of 4862F has not been previously reported in the literature. The trimethylated-Tyr residue has only been reported in publications a few times, and the dehydrated-His residue has not yet been reported.

Compound 4862F was further tested for the inhibition of HIV-1 protease, and the IC_50_ value is 15.26 nM (Table 3) based on an established model used for high-throughput fluorescence detection for HIV-1 protease substrate [4]. As the positive control, indinavir showed IC_50_ values of 4.6 nM. In contrast, 4862F did not show any inhibitory activity against the HIV-1 reverse transcriptase at 200 μg·mL^−1^, indicating the specificity of this compound.

**Table 3:** Raw data of inhibitory activity of 4862F on HIV-1 protease.

Lg (4862F, nM)	3.17	2.87	2.57	2.27	1.95	1.65	1.35	1.05	0.75	0.45
Mean of inhibition (%)	94.70	93.55	87.25	82.00	73.70	58.95	46.10	49.50	38.35	32.60

## 3. Experimental

### 3.1. General Methods

Column chromatography was performed with Diaion HP-20, ODS-A and Sephadex LH-20 (Mitsubishi Chemical Analytech Co., Ltd, Yokkaichi-shi, Mie, Japan). Semi-preparative HPLC was performed using an ODS column (Zorbax SB-C18 column; 5 μm; 9.4 × 250 mm; 2 mL·min^−1^; 280 nm). HPLC analysis was performed using an ODS column (Shimadzu C18 column; 5 μm, 4.6 × 150 mm; 1.0 mL·min^−1^; 280 nm) at 40 °C. ESI-MS data were acquired using a LTQ ORBITRAP XL mass spectrometer (Thermo-Fisher, Waltham, MA, USA). ^1^H-, ^13^C-NMR and DEPT spectra and 2D-NMR were recorded on a Bruker AV500-Ш spectrometer (Bremen, Germany) equipped with a 5 mm PABBO probe head, using TMS as internal standard and chemical shifts were recorded as δ values. The spectra were measured in CD_3_OD, and the chemical shifts were referenced to the residual solvent signal (δ_H_ 4.839, 3.251 and δ_C_ 48.997). ^1^H spectra were also measured in D_2_O and 2% *d*-TFA, and chemical shifts were referenced to the residual solvent signal (δ_H_ 4.800). Optical rotations were measured on a Perkin-Elmer model 343 polarimeter (Waltham, MA, USA).

### 3.2. Strain

*Streptomyces *sp*.* I03A-04862 was stored at the China Medicinal Microbiological Culture Collection Center (Institute of Medicinal Biotechnology, Chinese Academy of Medical Sciences and Peking Union Medical College, No. I03A-04862) at −80 °C.

### 3.3. Fermentation

*Streptomyces *sp*.* I03A-04862 was incubated in a rotary shaker (220 rpm) at 28 °C for 48 h in 500 mL Erlenmeyer flasks containing 100 mL medium consisting of glucose 0.5%, yeast extract 0.5%, peptone 0.5%, beef extract 0.5%, corn steep liquor 0.4%, soybean meal 1%, soluble starch 1.0%, CaCO_3_ 0.4%, and CoCl_2_·6H_2_O 0.002% in deionized water (pH 7.2 before sterilization) and then scaled up to 5,000 mL flasks containing 1,000 mL of the medium. The fermentations were carried out at 28 °C for 96 h with aeration and agitation.

### 3.4. Extraction and Isolution

The whole fermentation broth (30 L) was filtered to separate into supernatant and mycelia fractions. The mycelia were extracted three times with acetone (3 L). The acetone solution was evaporated under reduced pressure to afford an aqueous solution. The aqueous solution and the supernatant were subjected to Diaion HP-20 macroporous adsorbent resin column chromatography (3 L). A successive elution of the column with H_2_O and 30%, 50%, 75% and 100% acetone in H_2_O yielded five corresponding fractions (HP-0% ~ HP-100%) after removal of solvents. Fractions HP-30% and HP-50% (1 g), with 68.3% inhibitory activity on HIV-1 protease at 1 mg·mL^−1^ were purified by column chromatography using ODS-A (200 mL), and eluted successively with 40%, 50%, 60%, 80% and 100% aqueous methanol solution to yield five corresponding subfractions (ODS-40% ~ ODS-100%) after removal of solvents. The fractions ODS-50% and ODS-60% (93.4 mg, yield 9.3%) with 78.3% inhibitory activity on HIV-1 protease at 1 mg·mL^−1^ were separated by column chromatography using Sephadex LH-20 eluted with water (30.2 mg, dried power, yield 32.3%, with 90.3% inhibitory activity on HIV-1 protease at 1 mg·mL^−1^) and then subjected to reversed phase semi-preparative HPLC twice using a mobile phase of 45% methanol aqueous solution containing 0.1% trifluoroacetic acid (TFA) to yield 4862F (*t*_R_ 19.6 min, 8.5 mg, yield 28.2%, with 99.3% inhibitory activity on HIV-1 protease at 1 mg·mL^−1^).

### 3.5. Absolute Configuration

Solutions of 4862F (0.5 mg) in 6 N HCl (1.0 mL) were heated at 110 °C for 24 h. Upon removal of excess HCl under vacuum, the hydrolysates were placed in a 1 mL reaction vial and treated with a 1% solution of 1-fluoro-2,4-dinitrophenyl-5-L-alanine amide (FDAA, 150 μL) in acetone, followed by 1 N NaHCO_3_ (40 μL). The reaction mixtures were incubated at 45 °C for 1.5 h, cooled to room temperature, and then acidified with 2 N HCl (20 μL). Similarly, the standard L- and D-amino acids were derivatized separately. The derivatives of the hydrolysates and the standard amino acids were subjected to HPLC analysis using a mobile phase of 45% methanol aqueous solution containing 0.1% trifluoroacetic acid (TFA). The retention times for FDAA derivatives of the hydrolysates and the standard amino acids are summarized in Table 2.

### 3.6. Analysis of the Inhibition of HIV-1 Protease Activity by Compound 4862F

The assay method for HIV protease activity measurement is based on intramolecular fluorescence resonance energy transfer (FRET) developed by Edmund [5] and Dong [4]. The assay uses quenched fluorogenic substrates containing a peptide sequence derived from a natural processing site for HIV-1 protease. The quenched fluorogenic substrate (obtained from Sigma, St. Louis, MO, USA) was Arg-Glu (EDANS)-Ser-Gln-Asn-Tyr-Pro-Ile-Val-Gly-Lys-(DABCYL)-Arg, where the fluorescent donor is EDANS and the quenching acceptor is DABCYL. Recombinant HIV-1 protease was expressed in *E. coli* JM109 and purified to homogeneity [6]. Briefly, *E. coli* JM 109 (Novagen, Darmstadt, Germany) were transfected by a p100w plasmid coding for the corresponding enzyme. The insoluble recombinant protein, accumulated in the form of inclusion bodies, was isolated and solubilized in 8 M urea, 10 mM DL-dithiothreitol (DTT), and 20 mM Tris-(hydroxymethyl)-aminomethane (Tris, pH 8.0). The protease was purified by cation exchange chromatography using DEAE-sephacel and CM-cellulose (Sigma). Purified enzymes were stored at −85 °C.

Incubation of recombinant HIV-1 protease with the fluorogenic substrate at 37 °C resulted in specific cleavage of a tyrosine-proline bond, causing an increase in fluorescence due to elimination of intramolecular quenching. In the presence of an inhibitor such as indinavir, the fluorescence signal was expected to decrease as the concentration of inhibitor increased. Thus, inhibition of HIV-1 protease by 4862F in the presence of the fluorogenic substrate was observed as a decrease in fluorescence intensity. The data were obtained with a Polarstar spectrofluorometer (excitation at 340 nm, emission at 490 nm). All values were standardized with blanks. For all of the experiments, the protease was added last to keep the incubation time with inhibitor consistent.

## 4. Conclusions

A new inhibitor of HIV-1 protease, 4862F, has been isolated from the culture broth of *Streptomyces albosporus* I03A-04862, which was elucidated as *N,N,N-*(trimethylated)-Tyr-L-Leu-L-Val-L-Leu-(dehydrated)-His. This compound displayed inhibitory activity against HIV-1 protease, with a IC_50_ value of 15.26 nM using a fluorescence-based assay.
